# Face masks drive increased rational decision-making

**DOI:** 10.1007/s12144-022-03895-1

**Published:** 2022-11-04

**Authors:** Ramzi Fatfouta, Yulia Oganian

**Affiliations:** 1 HMKW Hochschule für Medien, Kommunikation und Wirtschaft, University of Applied Sciences, Ackerstraße 76, 13355 Berlin, Germany; 2 Center for Integrative Neuroscience, University Medical Center Tübingen, Ottfried-Müller-Str. 25, 72076 Tübingen, Germany

**Keywords:** COVID-19, Face masks, Decision making, Social cognition, Economic bargaining, Ultimatum Game

## Abstract

Face masks play a pivotal role in the control of respiratory diseases, such as the novel coronavirus (COVID-19). Despite their widespread use, little is known about how face masks affect human social interaction. Using unique experimental data collected early on in the pandemic, we investigate how facial occlusion by face masks alters socio-economic exchange. In a behavioral economics study (*N* = 481), individuals accepted more monetary offers and lower offer amounts when interacting with a masked versus unmasked opponent. Importantly, this effect was mainly driven by faces covered with surgical masks relative to bandana-type masks. In the first weeks of mask use during the COVID-19 pandemic, motive attributions further moderated this effect: Participants who believed that mask wearers were seeking to protect others showed the highest acceptance rates. Overall, we describe a new phenomenon, the face-mask effect on socio-economic exchange, and show that it is modulated by contextual factors.

## Introduction

The novel coronavirus (COVID-19) pandemic constitutes a public health crisis of unprecedented proportions. As of early October 2022, for example, more than 621 million total cases worldwide have been reported, with more than 6.5 million total deaths in over 188 countries (JHU, 2022). To curb the spread of the virus and, hence, to reduce its devastating effects, public-health experts and virologists have advised people to wear face masks in public places (for an overview, see Greenhalgh et al., 2020). Recent evidence indicates that mask wearing helps controlling the transmission of COVID-19 due to a reduction in potentially harmful droplets and aerosols (Howard et al., 2020; Leung et al., 2020; Mitze et al., 2020). Yet, research on how mask wearing affects human social interaction and, by extension, interactive decision-making is rather scarce – a gap we intend to close in the current paper.

In the present investigation, we experimentally address the important, albeit open, question of whether being exposed to masked faces affects economic decision-making in an iterated social exchange. Typically, people are advised to wear face masks when social distance cannot be maintained (e.g., in crowded places). Because face masks function as barrier gestures used to figuratively separate interaction partners from each other (Inglis, 2017), we reasoned that face masks might act as incidental cues of social distance (i.e., cues unrelated to the decision object at hand). This cued social distance, in turn, would give perceivers more room for rational decisions (or, less room for fairness concerns). Specifically, we demonstrate that face masks cause an increase in rational bargaining behavior, thereby enhancing participants’ monetary gains in social interactions. We further characterize the psychological and temporal dynamics of this novel face-mask effect and discuss potential implications for real-world behavior during the COVID-19 pandemic and beyond.

Given its increasing social acceptance in community settings (Feng et al., 2020), mask wearing in public likely influences individuals’ minds and behavior. Indeed, face masks occlude 50–60% of the face (i.e., nose, mouth, and chin; Hess, 2020), thereby impeding important social cues that guide our behavior (Zebrowitz, 2011). Recent research demonstrates that mask wearing impairs the reading of emotions and reduces confidence in judgments of facial expressions (e.g Carbon, 2020; Grahlow et al., 2022; McCrackin et al., 2022; Tsantani et al., 2022). Hence, aside from their potential benefits for disease prevention, face masks can also have important ramifications for everyday social behavior. Given the different interactive contexts that may be affected by mask wearing (e.g., grocery stores, public transport, and official buildings), understanding people’s responses to mask wearers in social contexts is of essential importance for ongoing public-health efforts regarding mask use.

Wearing a face mask in the current pandemic context is often interpreted as compliance to existing social-distancing measures. Yet, there are regional differences in the use of face masks before and during the pandemic (Bavel et al., 2020; Leone, 2020). East Asian countries (e.g., China, Korea, and Japan), for example, are more experienced with pandemics and resulting mask usage than Western countries (Feng et al., 2020; Miyazawa, 2020). Moreover, a recent study showed that compared to people in China people in Germany and Austria have a lower adherence to mask wearing during the COVID-19 pandemic (Zhao & Knobel, 2021). These findings make Western countries, including Germany and Austria, particularly suitable for investigating our research question because mask-wearing behavior is a relatively new behavior in these countries.

Over the course of the COVID-19 pandemic, the concepts of “mask wearing” and “distance” gradually became associated in people’s minds, especially among non-Asian people (Leone, 2020). Indeed, observational studies suggest that face masks may be a cue for others to keep a greater distance (Seres et al., 2020a, b). Moreover, Grundmann et al. (2021) showed that people perceive masked faces (vs. unmasked faces) to be more distant to themselves. Most relevant to our study, using implicit measures (i.e., the Implicit Association Test), Fatfouta and Trope (2021) demonstrated that people automatically associate masked faces (vs. unmasked faces) with psychological distance (i.e., the subjective experience that something is near or far away from the self). These findings support the view that face masks may act as incidental cues of distance, thereby holding the potential to affect people’s judgment and behavior.

In this article, we rely on game theory to address the role of face masks on decision making. Game theory is a collection of models that attempt to examine and explain situations in which the consequences of one player’s decision depend on the decision of other players. As such, game theory offers a wide variety of behavioral tasks (“economic games”) for the experimental investigation of social interaction (for an overview, see Gächter, 2004; Morgenstern & Von Neumann, 1953). Given previous research showing that face masks influence people’s judgments (e.g., Carbon, 2020), interactive games allow researchers to investigate the effects of mask wearing under controlled experimental conditions.

One of the most well-known games is the Ultimatum Game (Güth et al., 1982). In this game, one party (the proposer) is asked to split a certain amount of money with another party (the responder). If the responder accepts, the sum is divided as proposed. If the responder rejects, both players go away empty-handed. As predicted by rational choice theory (Simon, 1955), responders should maximize the expected utility of their profits and, hence, accept even the smallest amount of money proposed. Empirically, however, responders tend to deviate from rational choice and reject small, unfair, offers (i.e., ≤ 20% of the total amount). Such behavior illustrates that humans, on average, are willing to engage in so-called costly punishment to promote and maintain cooperation (Camerer, 2003).

Previous research on economic games shows that people’s fairness consideration is influenced by perceptions of social distance (Charness & Gneezy, 2008; Fatfouta et al., 2015). Kim et al. (2013), for example, demonstrated that when deciding for a stranger, as compared to deciding for oneself or a close other, individuals were more likely to favor economic utility by accepting unfair monetary offers. Similarly, it has been shown that unfair offers are more likely to be accepted from an out-group member than from an in-group member (Mendoza et al., 2014). Generally speaking, as social distance increases, “decision making is more likely to be guided by ‘cold’ processing” (Kim et al., 2013, p. 633). Hence, these findings support the view that distance cues help people to make more rational decisions. Based on these findings, we expected that face coverings could achieve a similar behavioral effect.

The main aim of the present study was to show that mask wearing is sufficient to cue the subjective experience of greater social distance between proposer and responder in ultimatum-game negotiations. As a result, responders should be able to overcome their immediate experience of being treated unfairly in the here and now and, instead, aim at maximizing their personal monetary payoffs. In this case, responders should manifest more rational bargaining behavior, which would be reflected in less costly punishment. Stated differently, if face masks cue social distance in people’s minds one should expect higher acceptance rates for unfair offers from mask-wearing proposers than from unmasked proposers (referred hereafter as *face-mask effect*).

Importantly, most of the aforementioned studies examining the effect of face masks only compared masked versus unmasked faces. From an experimental vantage point, however, this leaves open the possibility that the established effects are not specific to face masks but to other face coverings. Indeed, previous research examining different types of facial occlusions showed that face masks usually produce higher behavioral effects than other occlusions (Noyes et al., 2021). We therefore decided to include an additional control condition to examine the possibility that surgical masks are potentially more effective in promoting more deliberate and rational decision-making as compared to nonmedical community masks (i.e., “bandanas”).

Aside from establishing the face-mask effect, we also strived to shed light on potential boundary conditions under which face masks will trigger more rational decision-making. To address this issue, we sought to examine how motive attributions related to mask wearing may influence the face-mask effect on economic bargaining. According to attribution theory (Weiner, 1985), individuals attempt to understand the causes of others’ behaviors and such attributions in turn can modulate their reactions to those behaviors. Typically, people wear face masks for reasons of self- or other-protection (Feng et al., 2020). Observers therefore can attribute either selfish motives (i.e., the person wants to protect *themselves*) or altruistic motives to the mask wearer (i.e., the person wants to protect *others*). We thus sought to additionally explore whether the face-mask effect differs for individuals who would think that other people wear masks with an altruistic versus egoistic intent in mind.

### The Present Research: Aims and Predictions

The present research aimed to show that face masks affect people’s interactive decision-making. To accomplish this, a behavioral economics study among German-speaking participants using the Ultimatum Game examined the influence of two distinct face coverings, namely surgical masks and bandanas, on people’s bargaining behavior. We predicted that face masks (in particular, surgical masks) act as distance cues and thus let participants to accept more offers from mask-wearing opponents than from opponents without a mask. To further explore this face-mask effect, we additionally examined the role of motive attributions (altruistic vs. egoistic) on people’s decision behavior. Importantly, this will add to the studies referenced above on the psychological effects of mask wearing which mostly relied on observational or self-report methods instead of actual behavior.

## Methods

We report how we determined our sample size, all data exclusions (if any), all manipulations, and all measures in the study (Simmons et al., 2012). Complete data, code, and R output files used for the analyses reported below are available from the Open Science Framework (OSF; https://osf.io/d86yx/).

### Participants and Procedure

The study was conducted in Germany and Austria during late April and early June 2020 (April 29 – June 06). Our goal was to collect the largest data set possible in that time frame (see Power analysis section for further discussion of the sample size). Data collection was restricted to that period because mask wearing in public became mandatory in late April in these two countries and because lockdown restrictions were eased in early June. Moreover, in both countries mask use and policies were very similar at that time (i.e., face masks were required in public transport and stores).

A total of 481 German-speaking individuals were recruited via advertising on online social networks, mailing lists, or blogs to take part in a brief (10–12 min) online experiment on “social exchange during the COVID-19 pandemic”. As an incentive, participants were informed that they could earn actual money based on their decisions in the ultimatum game and that payoffs would be transferred via online banking after study completion.

Participants were excluded if they did not clearly understand the instruction (see instruction check questions as described below; *n* = 12; 2.5%), lived outside Germany or Austria (*n* = 2; 0.4%) because other countries may have different mask ordinances, and had a past or present diagnosis with COVID-19 (*n* = 66; 14%) because COVID-19-associated symptomatology has been shown to affect people’s neurocognitive ability (Lopez-Leon et al., 2021). Furthermore, for the experimental condition, we excluded individuals who were accustomed to mask wearing in their professional interactions, such as healthcare professionals (*n* = 67; 14.1%) because of their increased job-related familiarity with masked faces and because of prior research showing that such individuals may respond differently to decisions-making tasks (Mazza et al., 2020). The final sample thus consisted of 339 individuals (*M*_age_ = 26.93 years, *SD*_age_ = 8 years, age range = 18–72 years; 241 females). Most of the participants held a high school diploma (97%) and the majority was pursuing a university degree (72%). After providing informed consent, participants completed the materials described below. All materials were administered using a professional survey platform (SosciSurvey; www.soscisurvey.de; Leiner, 2019).

### Economic Bargaining

Participants obtained detailed instructions about the ultimatum game and answered two questions to check their understanding of the game rules (e.g., “If you accept the offer, how much money do you get?”). To increase realism, participants were informed that they would play the game in the role of the responder with several anonymous players who had submitted their offers in a previous study (for a similar procedure, see Mussel et al., 2013). Participants were further informed that their decisions would have an actual impact on both their individual payoff and their respective opponent. Yet, in reality, all offers were predefined to increase experimental control. Participants were fully debriefed at the end of the experiment.

**Fig. 1:**
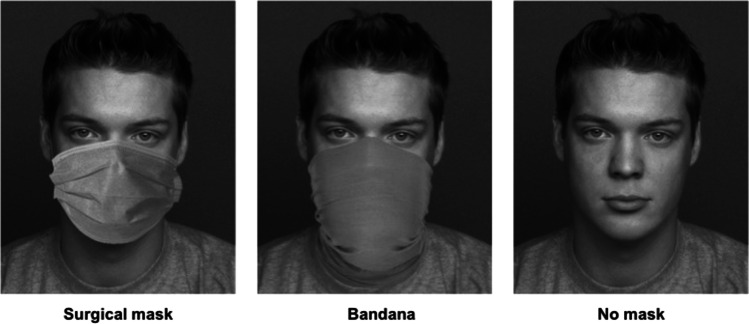
Examples of face stimuli used in the ultimatum game. From left to right, the presented faces illustrate an example proposer in the different experimental groups

We used a mixed factorial design, with the within-subject factor *fairness* (5 levels: offer magnitude of 1 to 5 out of 10 €), and the between-subject factor *mask* (3 levels: no mask, surgical mask, bandana cloth face mask). To note, *mask* was manipulated as between-subjects factor to eliminate the possibility that participants would be sensitized to our expectation of different decisions for different proposers that are either masked or not. We chose to focus on disposable surgical masks as our main experimental manipulation because they are most commonly used (Zuniga & Cortes, 2020). To examine whether facial occlusion by surgical masks exerts a unique effect on decision behavior, we additionally included another type of cloth face covering (i.e., bandana masks) that are not as strongly associated with the COVID-19 pandemic. The two mask conditions differed only in the type of face masks used and not in the degree of facial occlusion. See Fig. 1 for images of an example proposer in each experimental group, as presented on each trial.

In each experimental group (no mask, surgical mask, bandana cloth face mask), participants received one-shot monetary offers from 50 different proposers (in random order), each involving a 10 € split. Specifically, participants received equal proportions of 1:9, 2:8, 3:7, 4:6, and 5:5 € splits (offered: kept) from each proposer. Each round began with a central fixation cross (500 ms), followed by a picture of the proposer (evenly distributed across sex and matched for attractiveness) and the offer (4,000 ms). While the offer was presented, participants had to decide whether to accept or reject it (coded as: 0 = reject, 1 = accept). Immediately after response selection, the next trial started. If participants failed to respond on time, the game advanced automatically. Proposer pictures with a neutral facial expression were taken from the FACES database (Ebner et al., 2010). A professional graphic designer modified the pictures by individually applying face masks to cover the same part of each face. We used the same pictures without face masks for the control condition. All images were converted to grayscale.

### Perceptions of Fairness and Trustworthiness

Based on previous studies (Fatfouta & Trope, 2021; Seres et al., 2020a, b), we argue that face masks function as a social-distance cue. Yet, facial occlusion may also affect people’s trait perceptions of individuals wearing masks. For example, people who wear surgical masks might be seen as more fair or trustworthy, because surgical masks are intuitively associated with the medical profession. Different trait perceptions would thus represent an alternative explanation to the face-mask effect. To rule out this potential concern, in each experimental group, participants provided an overall evaluation of their opponents in the ultimatum game on perceived fairness (“How fair were the players during the game?”, 1 = *not at all fair*, 7 = *extremely fair*, *M* = 3.47, *SD* = 1.12) and trustworthiness (“How trustworthy were the players during the game?”, 1 = *not at all trustworthy*, 7 = *extremely trustworthy, M* = 3.87, *SD* = 1.32).

### Motive Attributions

Upon completion of the ultimatum game, participants also answered two questions that assessed motives for wearing a mask that are reflective of either a selfish intent (“This person is protecting themselves from my, possibly infectious, droplets”, 1 = *not at all*, 5 = *very much, M* = 2.66, *SD* = 1.25) or an altruistic intent (“This person is protecting me from their own, possibly infectious, droplets”, 1 = *not at all*, 5 = *very much, M* = 4.0, *SD* = 1.21).

### Additional Control variables

To additionally control for potential confounds associated with individual attitudes towards COVID-19, we assessed participants’ fear of COVID-19 using the 7-item fear of COVID-19 scale (sample item: “I am most afraid of coronavirus-19”, 1 = *strongly disagree*, 5 = *strongly agree*, *M* = 1.73, *SD* = 0.57, Cronbach’s α = 0.78; Ahorsu et al., 2020; German version: Fatfouta & Rogoza, 2021). Moreover, we assessed participants’ self-perceived risk of contracting COVID-19 using a single-item measure ("My self perceived risk of getting the coronavirus is…", 1 = *low-risk*, 7 = *high-risk*; *M* = 3.51, *SD* = 1.57; Harper et al., 2020). To further assess whether the continuous use of personal masks by the public (Feng et al., 2020) would affect their effect on social interactions, we also assessed the duration of personal mask use (assessed in weeks; *M* = 2.84, *SD* = 2.45, range = 0 – 13 weeks). For an overview of measures and zero-order correlations for the covariates, see Tables S1 and S2 (see Supplementary Online Material in the OSF).

### Data Analysis

We analyzed participants’ choices in a logistic mixed-effects model using the lme4 package in R (Bates et al., 2015). This approach allows concurrent modeling of within- and between-subject factors as well as continuous predictors at the population level. All models included the maximal random factor structure with random intercepts and slopes for the within-subject factor *fairness*. Overall significance of the categorical factor *mask* was assessed using type III Wald chi-squared (${\chi }^{2}$) tests. Significance of continuous predictors and single contrasts between levels of the factor *mask* was assessed using a *z*-approximation, as implemented in the lme4 package. 95% Confidence intervals for logistic mixed-effects models were calculated using the function ‘confint’ in R, using the Wald approximation. Model comparisons to assess the best model including covariates were done using BIC and a forward stepwise approach.

### Power considerations

Power in multi-level repeated-measures models depends on the number of observations per participant as well as the number of participants. Based on simulations in Brysbaert and Stevens (2018) with the SIMR package for power calculation in generalized mixed-effects models (Green & MacLeod, 2016), we estimated that in our design, with as few as 30 stimuli per condition from as few as 50 participants per condition (Maas & Hox, 2005), we had a power of at least 80% to observe a medium effect size (corresponding to Cohen’s *d* = 0.4 or larger) at α = 0.05 for the interaction between fairness and mask conditions. The obtained sample thus allowed estimating effects with sufficient precision.

## Results

### Preliminary Analysis: Fairness and Trustworthiness Perceptions

A potential concern could be that different masks might be associated with different trait perceptions. To rule out this alternative interpretation, we first examined whether fairness and trustworthiness perceptions differed among all three conditions, respectively (i.e., no mask, surgical mask, bandana cloth face mask). Importantly, we did not find any significant differences (all *p*’s > 0.5), indicating that the facial-occlusion manipulation did not affect people’s general trait impressions concerning fairness and trustworthiness.

### Main Analysis: The Face-Mask Effect on Economic Bargaining

**Fig. 2:**
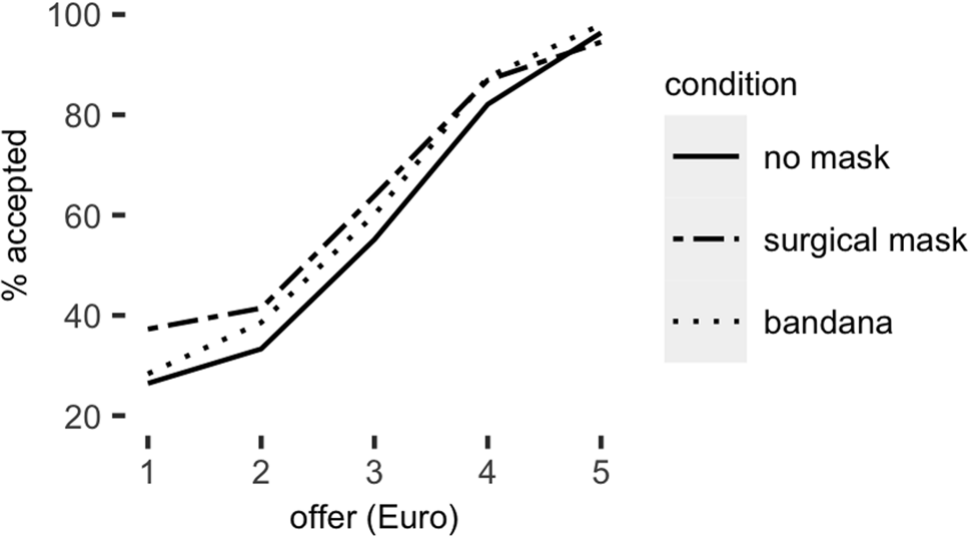
Ultimatum game decisions. Participants accepted significantly more unfair offers when their opponents’ faces were partially occluded with a surgical mask, but not when the face covering was a bandana cloth face mask

To test our overarching hypothesis that opponents wearing a face mask would alter responders’ offer acceptance rates, we tested the effects of group-level experimental factors fairness (1–5) and mask (no mask, surgical mask, bandana cloth face mask) on participants’ responses (Fig. 2). As expected, a significant main effect of fairness reflected that participants accepted more offers as they became fairer (beta = 3.10, *SD* = 0.25, ${\chi }^{2}$(1) = 155.65, *p* < 0.001, 95%CI = [2.62, 3.59]). This result is consistent with previous studies in the ultimatum-game literature (Güth et al., 1982; Kim et al., 2013; Mendoza et al., 2014).

More importantly, and as expected, we found a significant main effect of face covering (${\chi }^{2}$[2] = 7.15, *p* = 0.03), which was further qualified by a significant interaction effect with fairness (${\chi }^{2}$[2] = 7.1, *p* = 0.03). Consistent with our main prediction on the face-mask effect, participants were more accepting of unfair offers when interacting with a masked than unmasked person. Importantly, the face-mask effect was mainly driven by the surgical-mask condition (difference to no mask condition: beta = 4.18, *SD* = 1.6, *z* = 2.7, *p* = 0.008, 95%CI = [1.12, 7.24]; interaction with fairness: beta = -0.95, *SD* = 0.36, *z* = -2.62, *p* = 0.009, 95%CI = [-1.66, -0.24]): Participants accepted more unfair offers in the surgical-mask condition than in the control condition. In other words, when playing against opponents whose faces were occluded by a surgical mask, participants accepted more unfair offers, showing more rational behavior. In contrast, the bandana-mask condition did not differ significantly from the control condition (main effect *p* = 0.21, interaction *p* = 0.40). Overall, occlusion by a surgical mask had a unique effect on economic bargaining.

### Ancillary Analysis: Effects of Motive Attributions on Economic Bargaining

After having established the face-mask effect, we sought to further examine the potential role of COVID-19-related contextual variables. The difference between the surgical-mask condition and the bandana-mask condition suggests that the effect of surgical masks is specific to the type of face covering used and its associated role in the COVID-19 pandemic. To further examine how individual attributions about the motivation for mask use, individual fear of COVID-19, and self-perceived risk of contracting COVID-19 affect the effect of mask use on offer acceptance, we proceeded to test the effect of these variables in the surgical-mask condition. To this end, we fit logistic mixed-effects models to the data from the surgical mask condition only, including additional between-subject covariates: self-perceived risk of contracting COVID-19, fear of COVID-19, attributions of selfish intent (i.e., mask is worn for self-protection), and attributions of altruistic intent (i.e., mask is worn for other-protection). To further control for the possibility that these effects depend on the novelty of public mask use at that time, self-reported duration of mask use in public was added as an additional between-subject covariate.

Stepwise regression models revealed that the best model of offer acceptance in the surgical-mask condition included the duration of mask use and attributions of altruistic intent. As expected, this model also revealed a significant main effect of fairness with fair offers being more frequently accepted than unfair offers (beta = 2.03, *SD* = 0.26, *z* = 7.7, *p* < 0.001, 95%CI = [1.52, 2.55]). Moreover, a two-way interaction of duration of mask use and belief that mask use is altruistic reflected that, at the beginning of mask use, participants accepted more offers when they believed that others wear masks with an altruistic intent in mind, but that this effect was attenuated with prolonged mask use (beta = -2.08, *SD* = 0.62, *z* = -3.34, *p* < 0.001, 95%CI = [-3.29, -0.86]). With time, however, all participants accepted more offers from opponents wearing a surgical mask, independent of the attributed motive. Additionally, the three-way interaction of this effect with offer fairness showed that this effect was more pronounced for unfair offers than for fair offers (beta = 0.67, *SD* = 0.32, *z* = 2.09, *p* = 0.036, 95%CI = [0.04, 1.31]). None of the other covariates improved model fit.

**Fig. 3:**
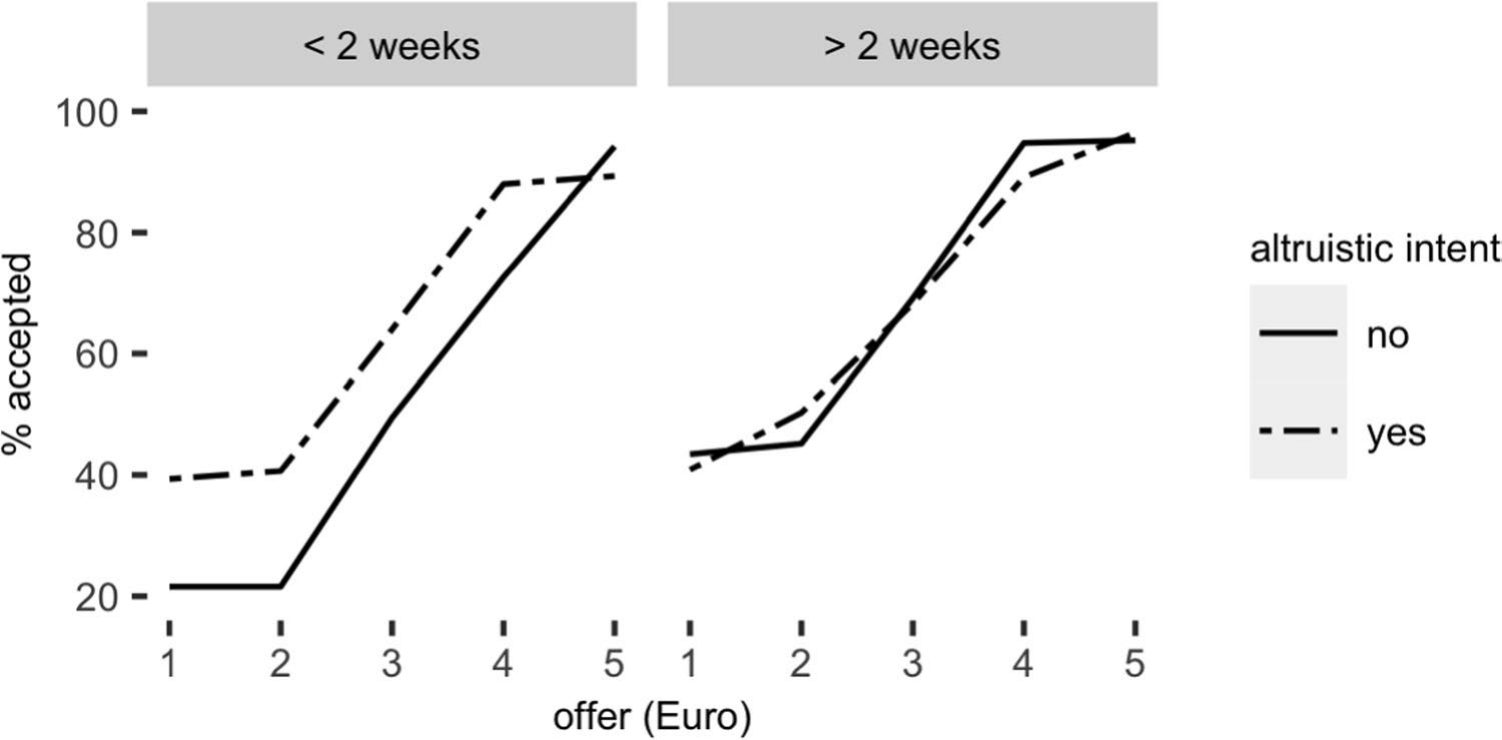
Effect of duration of mask use and attributions of altruistic intent for mask use on offer acceptance. In initial weeks of mask use, only participants that believed that mask wearers protect others, accepted more unfair offers. With prolonged mask use, participants accepted unfair splits at a higher rate, independently of individual beliefs about others’ motivation for mask use. Median split of continuous variables for illustration purposes only

Of note, the covariate analyses were conducted specifically within the surgical-mask condition, because this was the one condition where our manipulation affected decision-making behavior. To validate that the effects of motive attribution and duration of mask use were specific to the surgical mask condition, we also tested the effects of these covariates within the full model, including all three conditions (i.e., no mask, surgical mask, bandana cloth face mask). In this model, we found a significant three-way interaction between duration of mask use, altruistic intent and the contrast between the surgical mask and the no mask conditions, ${\chi }^{2}$[2] = 11.48, *p* = 0.003. This result confirms that the effects of duration of mask use and attributions of altruistic intent are specific to the surgical-mask condition (triple interaction for surgical mask vs. no mask contrast: beta = -2.42, *SD* = 0.7, *z* = -3.4, *p* < 0.001, 95%CI = [-3.81, -1.03]). Furthermore, none of these effects were significant in the control and bandana-mask conditions. In sum, we found that the face-mask effect can be modulated by additional contextual variables (i.e., duration of mask use and motive attributions), pointing to important temporal and psychological dynamics of this effect (Fig. 3).

## Discussion

Wearing face masks in the community can help slowing the spread of respiratory diseases such as COVID-19 and protecting risk groups from infection. Here, we report how mask wearing affects a vital domain of human social interaction, namely economic decision-making. We hypothesized that face masks (in particular, surgical masks) which typically function as barrier gestures might act as incidental cues of social distance, thereby leading individuals to become more rational in their bargaining behavior (i.e., focusing on global utility maximization vs. immediate fairness concerns). Using the ultimatum game as an experimental paradigm to investigate social exchange, we found evidence in favor of our main hypothesis, which we term the face-mask effect.

We obtained the following three main results. First, acceptance rates were increased in general when the opponent wore a face mask and this effect was more pronounced for unfair offers. Of particular importance, we also established that these results were dependent on the type of face covering used (i.e., surgical masks). Second, individual differences in altruistic (but not selfish) motive attributions modulated the face-mask effect to the extent that acceptance rates for unfair offers were higher when participants believed that others wear masks due to an other-regarding, selfless motive. Third, with extended mask use, acceptance rates were increased independent of attributed motives. Finally, we showed that these results persist even when controlling for COVID-19-related thoughts and feelings (i.e., fear of COVID-19 and self-perceived risk of contracting COVID-19).

Recent empirical findings show that when parts of a face are occluded by a mask people perceive the face to be more distanced from themselves although the objective distance from the self remains unaltered (Fatfouta & Trope, 2021; Grahlow et al., 2022). Extending these findings, our findings show that these perceptions can modulate people’s other-regarding behavior. More broadly, our research suggests that task-irrelevant incidental cues, namely, face masks, affect people’s fairness considerations in an economic bargaining situation. Importantly, our research highlights that mask wearing has consequential behavioral implications beyond their primary utility in preventing the spread of viruses in a pandemic context.

Our results suggest that surgical masks, as compared to more casual non-medical cloth face coverings (e.g., bandanas), are more effective in affecting people’s bargaining behavior. This result is consistent with recent research demonstrating an advantage for surgical masks over alternative face coverings, suggesting that their effect goes beyond just occluding parts of the face (Hies & Lewis, 2022). One potential explanation for the different pattern of results for the different face coverings is that people might be less familiar with viewing surgical masks on a face than viewing bandanas on a face. Indeed, surgical masks (e.g., disposable sterile medical masks) are typically worn by healthcare professionals in highly formal contexts (e.g., hospitals), whereas bandanas (e.g., casual bandana-type masks) are also worn as a fashion accessory in our day-to-day life. This interpretation is consistent with the meaning of surgical masks in Western countries before the pandemic. Accordingly, people make the experience of surgical masks only in specific circumstances, for example, when enduring “traumatic interventions on the body” (Leone, 2020, p. 1). Another, more parsimonious explanation is that over the course of the COVID-19 pandemic, surgical masks became strongly associated with the concept of distance, because they should be worn when physical distancing is not possible.

More broadly, our results align with previous behavioral economics research on social-distance effects (Kim et al., 2013; Mendoza et al., 2014). Extending these findings to the current COVID-19 pandemic, we show that even a facial manipulation (i.e., wearing a surgical mask) is sufficient to create such effects. Furthermore, it is especially interesting that attributions of an altruistic intent were crucial for motivating participants to accept more offers from masked opponents. This finding gives empirical support to anecdotal evidence that mask wearing may be seen as a “symbol of social solidarity” (Cheng et al., 2020, p. 2). One explanation for why selfish motives were not effective in modulating participants’ behavioral reactions could lie in the fact that most authorities stress the protection of others (and not oneself) as a prime reason for mask wearing in public (Greenhalgh et al., 2020), thus making altruistic over egoistic motives more salient in people’s minds.

Crucially, the modulating effect of motive attributions was more pronounced for individuals who reported using masks for a short period of time (i.e., less than two weeks), suggesting that attributions of an altruistic intent are especially time-sensitive. With more time and, hence, greater familiarity with face masks, motive attributions seem to become less relevant to individual decision making. This result agrees with the observation that as the level of familiarity with an attitude object increases, decision makers are less likely to make dispositional attributions about others (Burger & Pavelich, 1994; Nisbett et al., 1973; Shaver, 2016). Accordingly, in the context of the current study, the more participants were used to mask wearing in public, the more familiar they should be with mask-wearing faces, and the less likely they might rely on dispositional attributions as causal explanations for the proposer’s behavior. Thus, our finding that the face-mask effect still persists after two weeks of mask wearing suggests that these attributions fade away and, instead, may be replaced by a notion of togetherness and community (Shen, 2020).

### Potential Limitations and Directions for Future Research

Despite being the first study to describe the face-mask effect on economic decision-making, our results and their implications need to be interpreted with caution. In the current study, no actual social interaction was investigated, but one specific aspect of resource sharing in an economic game. We chose to focus on economic decision-making because it requires people to make consequential decisions about monetary gains and losses (i.e., high-stakes decisions). In future research, however, it might be worthwhile to examine how face masks affect real-world decision-making processes, such as purchasing decisions, risk taking, negotiations, and a plethora of other decision behaviors.

Our experiment was conducted in Germany and Austria at a time when social distancing requirements, respiratory etiquette, and mask wearing were rather new in place. While this time frame offers a unique opportunity to examine the face-mask effect on decision making and motive attributions, an open question is the generalizability of our findings across time and countries. Meanwhile, most people are widely familiar with wearing and perceiving face masks in different social contexts. Hence, our work provides a starting point for future longitudinal work that might examine the malleability of our effects across the different waves of the COVID-19 pandemic. Future studies are also warranted to scrutinize the effects of mask wearing, motive attributions, and individual behavioral choices in other countries affected by the COVID-19 pandemic, including the need to examine the impact of policies and cultural norms on mask wearing. Specifically, mask-wearing recommendations differ across health authorities worldwide (Feng et al., 2020). In most Scandinavian countries (e.g., Norway, Sweden, and Finland), unlike in almost all other Western European countries, residents are not required to wear face masks by federal law. In contrast, in many Asian countries (e.g., China, Japan, Taiwan) mask wearing in public is common practice, especially during flu season (Feng et al., 2020). Due to these partly discrepant regulations, future work is warranted to investigate to what extent the face-mask effect also occurs when people are hardly or very strongly accustomed to masks. Furthermore, examining the trajectories of our effects with even more extended mask use will be particularly interesting in light of existing country-specific differences.

Finally, although we were able to rule out two potential alternative explanations of our results (i.e., rejection of unfair distributions due to individual differences in fear of COVID-19 and/or self-perceived risk of contracting COVID-19), more research is needed to further substantiate the distance-cueing mechanism we propose. For example, it might also be that surgical masks prime associations with the medical profession and, hence, more respect for people with those jobs (especially right now), which then could result in a lack of willingness to punish those people (for an extended discussion, see Klucarova, 2022). Yet, such professional/medical cue priming effect should presumably affect trustworthiness and fairness perceptions as well, which was not the case in our study. Indeed, the face coverings did not affect such trait perceptions of the opponents. This might change, however, when using a within-subjects design when different face coverings can be compared against each other. Future work is needed to examine how this experimental variation affects people’s decision-making behavior.

To conclude, there is lack of research on the psychological effects of mask wearing on human social interaction. Our study represents a first step towards understanding the impact of face masks on a consequential social domain, namely, economic bargaining. Our results showcase that face masks may not only limit the spread of viral diseases but have non-negligible behavioral effects.

## Declarations

### Data availability

Complete data, code, and R output files used for the analyses reported below are available from the Open Science Framework (https://osf.io/d86yx/).
